# Age-associated photoplethysmographic features across systolic blood pressure strata: a cross-sectional study

**DOI:** 10.3389/fphys.2026.1992759

**Published:** 2026-10-29

**Authors:** Chin-Nan Lin, Jen-Jyh Lin, Chun-Cheng Wang, Chih-Ping Chang, Yen-Ching Hung, Li-Chun Wang, Po-Yen Ko, Li Jingling

**Affiliations:** 1 Graduate Institute of Biomedical Sciences, China Medical University, Taichung, Taiwan; 2 Department of Chinese Medicine, Dalin Tzu Chi Hospital, Buddhist Tzu Chi Medical Foundation, Chiayi, Taiwan; 3 China Medical University, Taichung, Taiwan; 4 Department of Medicine, China Medical University Hospital, Taichung, Taiwan; 5 CardiAxis Technology Company Limited, Taichung, Taiwan

**Keywords:** aging, harmonic analysis, photoplethysmography, pulse rate variability, systolic blood pressure, waveform sharpness

## Abstract

Age-related vascular changes may alter photoplethysmographic (PPG) waveform morphology, timing, harmonic composition, and pulse rate variability. However, no evidence confirms that these age-associated PPG characteristics are consistent across different systolic blood pressure (SBP) levels. This cross-sectional study descriptively compared PPG-derived features between adults aged <65 and ≥65 years across four measured SBP strata and evaluated their associations with continuous age using multivariable regression. We included 1,163 cardiology outpatients. Within each stratum, age group differences were evaluated using the Mann–Whitney U test. An exploratory intersection analysis identified 11 candidate PPG-derived features showing nominally significant age-group differences across all four strata. After within-stratum FDR correction across all screened features, 10 of these candidates retained significance across all four strata. Older adults exhibited higher normalized waveform width indices, prolonged systolic timing, higher systolic to diastolic duration ratio, lower H2/H1 and low/high frequency ratios, and greater variability in pulse wave area than younger adults. Participants with SBP ≤120 mmHg also demonstrated these patterns. The findings identify a set of candidate age-associated PPG features consistently observed across the measured SBP spectrum. After adjustment for measured SBP, sex, body mass index, selected cardiometabolic comorbidities, and antihypertensive medication use, these features remained significantly associated with continuous age. Therefore, changes in waveform morphology, pulse timing, harmonic composition, and PRV-derived spectral characteristics may remain age-related. However, further validation remains necessary.

## 1 Introduction

Hypertension is one of the most significant global health challenges, affecting more than one billion individuals worldwide and serving as a principal driver of cardiovascular morbidity and mortality. Elevated brachial systolic blood pressure (SBP) is a clinical marker of hypertension, but it reflects only a small portion of the arterial system’s underlying hemodynamic burden (NCD Risk Factor Collaboration (NCD-RisC), 2021). Traditional cuff-based blood pressure measurement provides only intermittent, momentary readings, unable to capture the complex, beat-to-beat vascular dynamics contributing to cardiovascular risk (Parati et al., 2015). Consequently, many individuals, particularly older adults, exhibit substantial vascular dysfunction despite appearing normotensive according to office SBP measurements (McEniery et al., 2005). Considering this disconnect between brachial pressure and true vascular load, physiologically sensitive tools that can detect early arterial aging are urgently needed.

Aging profoundly alters the arterial tree’s structural and functional properties. Collectively, pathophysiological changes such as elastin loss, collagen accumulation, endothelial impairment, and medial thickening accelerate pulse wave velocity (PWV) and increase late systolic load (O'Rourke and Hashimoto, 2007). These alterations may also shorten diastole and increase pulsatile energy delivered to microvascular beds. Generally, they precede overt hypertension and independently contribute to heart failure with preserved ejection fraction (HFpEF), chronic kidney disease (CKD), and cerebrovascular dysfunction (Mitchell et al., 2010). However, routine clinical evaluation cannot fully elucidate these hemodynamic processes, with current guidelines predominantly relying on brachial SBP to define risk categories. This limitation is particularly problematic in older adults, who are likely to experience substantial vascular stiffening and wave reflection abnormalities despite having SBP within normal limits (Franklin et al., 1997).

Photoplethysmography (PPG), a simple and noninvasive optical technique for measuring blood volume changes in the microcirculation, is a powerful tool for characterizing vascular function (Allen, 2007). Aside from its utility in pulse oximetry and wearable technologies, PPG offers beat-to-beat morphological and temporal signatures reflecting arterial stiffness, wave reflection, autonomic regulation, and microvascular load (Takazawa et al., 1998). According to our prior work and other recent studies, PPG-derived indices such as normalized waveform geometry, harmonic energy distribution, systolic timing, and pulse-rate variability have been associated with cardiovascular physiology and measured SBP in previous studies (Lin et al., 2025). While our previous study primarily examined associations between comprehensive PPG features and SBP categories, the current one addresses a distinct research question by focusing on age-associated PPG differences within measured SBP strata rather than on SBP classification alone.

Subtle hemodynamic changes identified among older adults carry disproportionate clinical consequences. Even small increases in arterial stiffness or systolic load can induce microvascular injury, impair coronary perfusion, and exacerbate hypertension risk (Laurent and Boutouyrie, 2007). By understanding which PPG-derived features consistently differentiate younger from older individuals across several SBP categories, clinicians can identify early vascular deterioration more effectively and further elucidate age-specific mechanisms underlying hypertension development.

To address this question, this study aimed to compare PPG-derived features between adults aged<65 years and those aged ≥65 years within four measured SBP strata: ≤120, 121–139, 140–159, and ≥160 mmHg. This SBP-stratified analysis was an exploratory approach aimed at identifying PPG characteristics that show consistent age group differences across the SBP spectrum.

In addition, we aimed to evaluate whether the identified candidate features remained associated with age after accounting for measured SBP and selected clinical covariates. PPG features reflecting systolic waveform geometry, pulse timing, harmonic composition, and PRV-derived spectral characteristics were hypothesized to demonstrate age-associated changes beyond those explained by measured SBP alone.

## 2 Materials and methods

### 2.1 Study design and participants

This cross-sectional study was conducted at China Medical University Hospital, Taichung, Taiwan. Consecutive adults attending the cardiology outpatient clinics were recruited without targeted enrollment, matching, or quota sampling according to age or SBP. Eligible participants were as follows: aged ≥20 years, provided written informed consent, and remained seated at rest for at least 10 min before blood pressure and PPG measurements. The exclusion criteria were as follows:

documented cardiac arrhythmias, including atrial fibrillation or atrial flutter;

presence of an implanted permanent pacemaker;<15 s interval between repeated measurements;

ongoing maintenance dialysis;

documented severe peripheral arterial disease that was considered likely to compromise peripheral perfusion or reliable finger PPG acquisition;

structural digital or carpal abnormalities interfering with proper sensor placement;

incomplete or excessively noisy PPG recordings after preprocessing; and

PPG segments with a signal quality index (SQI) of<0.2%.

Of note, participants were not recruited according to predefined age or SBP quotas. After enrollment and signal quality assessment, participants were retrospectively classified according to their measured SBP, as described below. Within each SBP stratum, participants were categorized into two age groups (<65 and ≥65 years) for descriptive and exploratory age-group comparisons. The 65-year threshold was selected *a priori* because it is widely used in clinical and epidemiological research as a conventional boundary for older adulthood, rather than being derived from the present data. Continuous age, rather than dichotomized age, was used as the primary exposure in the inferential multivariable analyses.

The present cohort represents an expansion of our previously reported cohort. All 590 participants included in our prior SBP-focused study (Lin et al., 2025) were included within the current cohort of 1,163 participants, reflecting continued recruitment under the same study protocol. Importantly, the present analysis addresses a distinct research question focused on age-associated PPG characteristics within measured SBP strata rather than differences across SBP categories themselves. For overlapping participants, the original PPG recordings were reprocessed using the current analysis pipeline to ensure consistent signal processing and feature extraction across the entire cohort.

**Figure 1** summarizes participant enrollment and exclusions, retrospective SBP classification, age group categorization, and the subsequent analytical workflow. Based on the exclusion and signal quality criteria, 1,163 of 1,387 candidates were included in the final analysis.

**Figure 1:**
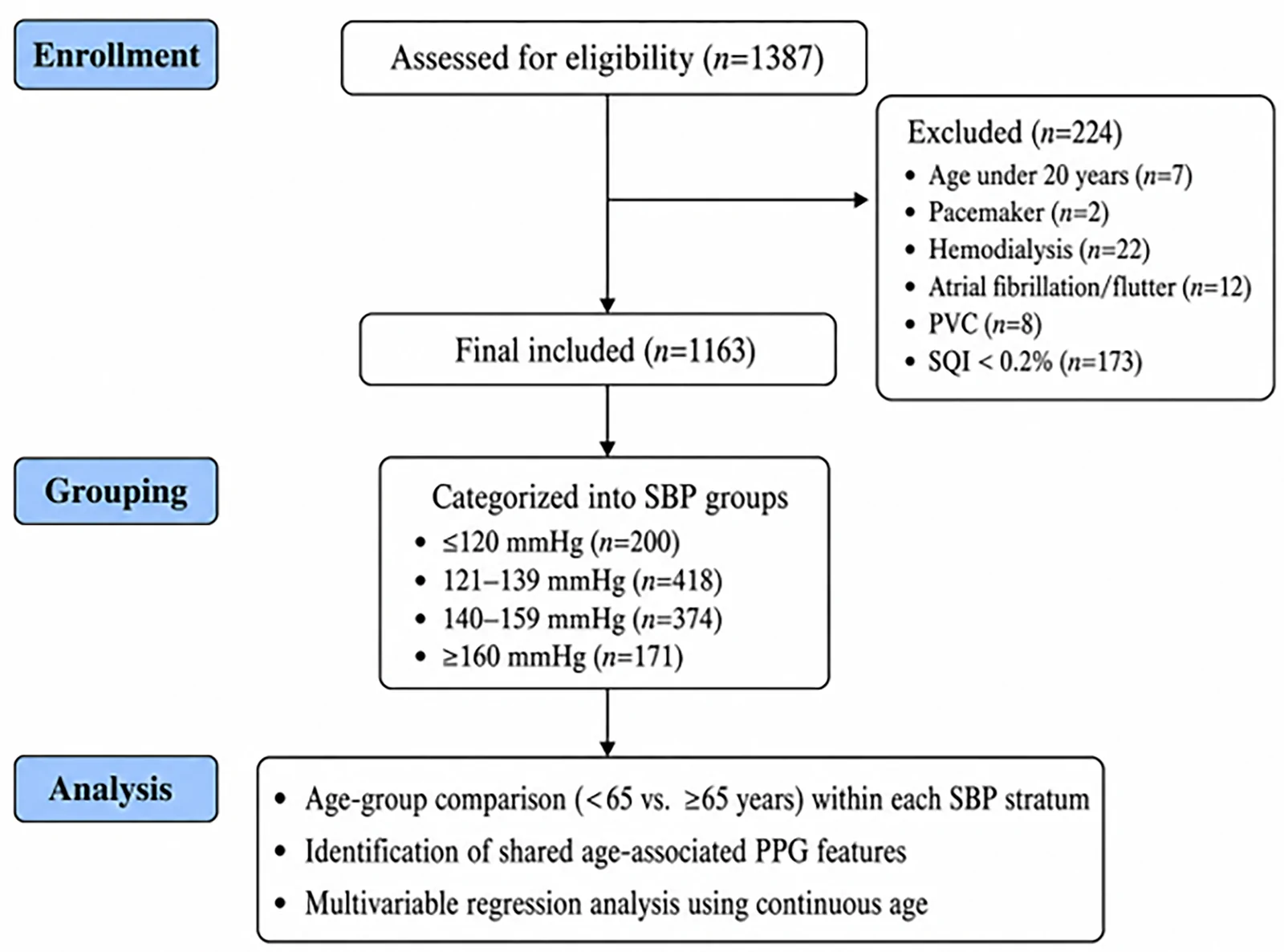
Flowchart of participant enrollment and exclusions, retrospective SBP classification, age group comparison, shared age-associated PPG feature identification, and multivariable regression analysis. PVC,; SQI, signal quality index; SBP, systolic blood pressure; PPG, photoplethysmography.

### 2.2 Blood pressure measurement and grouping

A validated automated oscillometric sphygmomanometer (HBP-9030, Omron Healthcare Co., Ltd., Kyoto, Japan) was used for measuring blood pressure. Participants remained seated at rest for at least 10 min before measurement, with the right arm supported at the heart level. Two blood pressure measurements were obtained at 1-min intervals. We used the mean values for SBP and diastolic blood pressure for the analysis.

After enrollment and quality control procedures, participants were retrospectively classified into four SBP strata according to their mean measured SBP: ≤120, 121–139, 140–159, and ≥160 mmHg. These categories reflect clinically interpretable SBP ranges while maintaining adequate sample sizes for age group comparisons within each stratum. The SBP strata were based on measured blood pressure at the study visit, thereby not representing formal diagnostic categories of hypertension.

The SBP strata were not defined by hypertension history and antihypertensive treatment status. Therefore, the lowest SBP stratum may have included both untreated participants with measured SBP ≤120 mmHg and patients with pharmacologically controlled hypertension.

### 2.3 Ethical considerations

The Research Ethics Committee III of China Medical University and Hospital reviewed and approved the study protocol (approval no.: CMUH114-REC3-111). This study obtained written informed consent from all participants and adhered to the principles of the Declaration of Helsinki.

### 2.4 Signal processing and quality control

The methods for PPG acquisition and signal conditioning, adapted from our previous study (Lin et al., 2025), were further modified to strengthen waveform reliability. The PPG acquisition system used in this study is associated with Taiwan Utility Model Patent No. 114206342, held by CardiAxis Technology Company Limited. Chin-Nan Lin and Yen-Ching Hung are inventors of this patent. PPG signals were collected using a MAX30102 optical sensor module (Maxim Integrated, USA) connected to Raspberry Pi 5 (Raspberry Pi Foundation, UK) via the I^2^C bus. Each participant underwent a continuous 90-s recording session (sampled at 500 Hz) from the left index finger. In all measurements, participants were in a seated resting state under minimized ambient illumination to reduce optical noise and external light contamination.

A structured multistage pipeline was used for signal preprocessing. First, raw infrared (IR) waveforms were band-pass filtered between 0.5 and 8 Hz using a finite impulse response filter with a Kaiser window to reduce motion- and respiration-related artifacts while preserving physiologic pulsatile components (Liang et al., 2018). Low frequency (LF) drift was further removed using a 1.5-s moving-average baseline correction. Before feature extraction, waveform scales were standardized across all participants through amplitude normalization.

To evaluate waveform reliability, we calculated SQI for each segment as the ratio of the pulsatile alternating current (AC) component to the static direct current (DC) component of the IR signal as follows:


$$SQI=\frac{AC}{DC}\times 100\%$$

The AC component was defined as the peak-to-peak amplitude of the filtered IR signal, calculated as follows: max (filtered IR) − min (filtered IR).

Conversely, the DC component was the mean value of the corresponding raw IR signal. This ratio conceptually corresponds to the perfusion index commonly used in PPG studies and reflects the strength of the pulsatile component relative to the static optical background (Goldman et al., 2000; Orphanidou et al., 2015).

A low pulsatile-to-static ratio frequently indicated insufficient peripheral perfusion, motion interference, peripheral vasoconstriction, or unstable waveform morphology; thus, segments with SQI< 0.2% were excluded. Meanwhile, we retained segments with SQI ≥ 0.2% for feature extraction. The 0.2% threshold was selected as a conservative lower bound based on the preliminary assessment of waveform stability and feature reproducibility in our dataset (Elgendi, 2016).

Systolic peaks referred to local maxima of the filtered waveform after derivative-assisted zero-crossing validation, consistent with previously described fiducial definitions (Elgendi et al., 2013). Beat-to-beat intervals were then calculated from consecutive systolic peaks to obtain pulse rate and pulse rate variability metrics.

### 2.5 Feature extraction

We extracted a structured set of PPG features from each 90-s recording to characterize waveform morphology, timing behavior, harmonic composition, and pulse rate variability. Our previously developed methodology, applied and validated in clinical signal analyses, was the basis of the overall feature framework.

#### 2.5.1 Time-domain morphology and rhythm features

On the filtered IR waveform, key fiducial landmarks, including the systolic peak, secondary (diastolic) peak, valley points, and dicrotic notch, were identified to quantify pulse morphology. We then computed temporal descriptors (e.g., systolic time, diastolic time, systolic–diastolic interval ratio, pulse amplitude slopes, and pulse area) from these landmarks. Ts was defined as the time interval from the preceding pulse valley to the systolic peak, whereas Td was defined as the interval from the dicrotic notch to the subsequent pulse valley. Ts/Td was calculated as the ratio of the mean Ts to the mean Td. These timing features were derived using the same underlying fiducial-point framework as in our previous study, while all recordings in the present cohort, including overlapping recordings, were processed using the current pipeline.

STD_AREA was defined as the standard deviation of beat-to-beat pulse area values across the analyzed recording.

To characterize rhythmic fluctuations in pulse timing, we calculated beat-to-beat variability measures—including the standard deviation of normal-to-normal beat intervals (SDNN), root mean square of successive differences (RMSSD), percentage of the number of normal-to-normal beat intervals that differ by >50 ms (pNN50), and pulse rate.

#### 2.5.2 Waveform geometry and sharpness indices

Systolic waveform geometry was characterized at multiple amplitude levels using six normalized waveform width indices (previously termed waveform sharpness indices): 1_10, 1_8, 1_6, 1_5, 1_3, and 1_2. For each pulse cycle, the horizontal waveform width was measured at predefined fractional heights of the systolic peak and normalized to the corresponding beat duration. Therefore, a higher value represents a broader normalized waveform contour at the specified amplitude level, whereas a lower value represents a narrower contour. They were defined and computed according to our previously published methodology (Lin et al., 2025).

#### 2.5.3 Frequency-domain and PRV-derived spectral indices

Fast Fourier transform with Welch averaging was used to derive frequency-domain features. Harmonic energy ratios (H1–H4) were extracted to describe waveform energy distribution across the fundamental and higher-order components, mirroring forward-propagating and reflected pulse waves.

To characterize frequency-domain pulse rate variability, LF and high-frequency (HF) spectral components and the LF/HF ratio were derived from the pulse interval variability spectrum.

Pulse intervals were calculated from consecutive systolic-peak times and assigned to the midpoint between adjacent peaks. The irregularly sampled pulse-interval series was resampled at 8 Hz using spline interpolation and detrended before spectral analysis. Welch power spectral density estimation was performed using a Hamming window with 50% overlap. The window length was set to the smaller of 256 samples or one-half of the interpolated series length and was constrained to an even number. The FFT length was defined as the larger of 1024 points or the next power of two greater than or equal to the interpolated series length. For a typical 90-s recording, this corresponded to a 256-sample (32-s) window and a 1024-point FFT, with an FFT frequency-bin spacing of approximately 0.0078 Hz. LF and HF power were integrated over 0.04–<0.15 Hz and 0.15–0.40 Hz, respectively, and LF/HF was calculated as the ratio of the corresponding integrated powers. No additional pulse-interval outlier correction was applied within the PRV spectral routine beyond the preceding signal-quality filtering and systolic-peak validation procedures.

Because these measures were obtained from peripheral PPG rather than ECG, they were interpreted as PRV-derived spectral indices rather than direct measures of cardiac autonomic balance.

#### 2.5.4 Quality-controlled feature computation

All feature calculations were limited to waveform segments with an SQI ≥ 0.2% to incorporate only stable and physiologically valid pulse segments, thereby enhancing the reliability of derived features and reducing the influence of noise or motion artifacts.

### 2.6 Statistical analysis

All statistical data were analyzed using Python (version 3.10). Continuous variables, summarized as mean values, were compared between the<65- and ≥65-year groups within each SBP stratum using the Mann–Whitney U test, given that several PPG-derived features were nonnormally distributed. Rank-biserial correlation was calculated as the effect-size measure for each Mann–Whitney U comparison, with positive values indicating higher values in the ≥65-year group and negative values indicating lower values in the ≥65-year group. A total of 27 PPG-derived features were evaluated in the initial within-stratum screening analysis. A nominal two-sided P< 0.05 threshold was used for the exploratory screening and subsequent intersection analysis. To assess robustness to multiple testing, Benjamini–Hochberg false discovery rate correction was additionally applied separately within each SBP stratum across all 27 features. The nominal intersection analysis was retained for exploratory candidate-feature identification, whereas the FDR-adjusted results were considered a multiplicity-adjusted sensitivity analysis.

Feature behavior across strata was summarized by tabulating the mean values and corresponding P-values for the 11 PPG features in each SBP group. Trends were not formally compared across SBP strata because we primarily aimed to characterize age-related differences within each SBP category rather than to evaluate between-stratum trends.

To evaluate whether the 11 candidate PPG features remained associated with age after accounting for measured SBP and clinical covariates, separate multivariable linear regression models were constructed for each feature. Age was modeled as a continuous independent variable. Each model was adjusted for measured SBP, sex, body mass index (BMI), diabetes mellitus (DM), coronary artery disease (CAD), congestive heart failure (CHF), chronic kidney disease (CKD; estimated glomerular filtration rate<60 mL/min/1.73 m^2^), and antihypertensive medication use, including calcium channel blockers (CCBs), angiotensin-converting enzyme inhibitors or angiotensin receptor blockers (ACEIs/ARBs), β-blockers, α-blockers, and thiazide diuretics.

For visualization of the age-group differences across measured SBP strata (**Figure 2**), separate multivariable linear models were fitted for each of the 11 candidate PPG features using categorical age group (<65 vs. ≥65 years), categorical SBP stratum, and their interaction, together with measured SBP, sex, body mass index, diabetes mellitus, coronary artery disease, congestive heart failure, chronic kidney disease, and antihypertensive medication use as covariates. These models were distinct from the primary regression models evaluating continuous age associations. Covariate-adjusted estimated marginal means (EMMs) were calculated for each age-group × SBP-stratum combination, with the remaining covariates fixed at their observed sample means. Model-based 95% confidence intervals were calculated for the adjusted estimates. The models were fitted using the original analytical cohort of 1,163 participants, with feature-specific exclusion of one participant with invalid Ts and Ts/Td values from the corresponding timing-feature models only.

**Figure 2:**
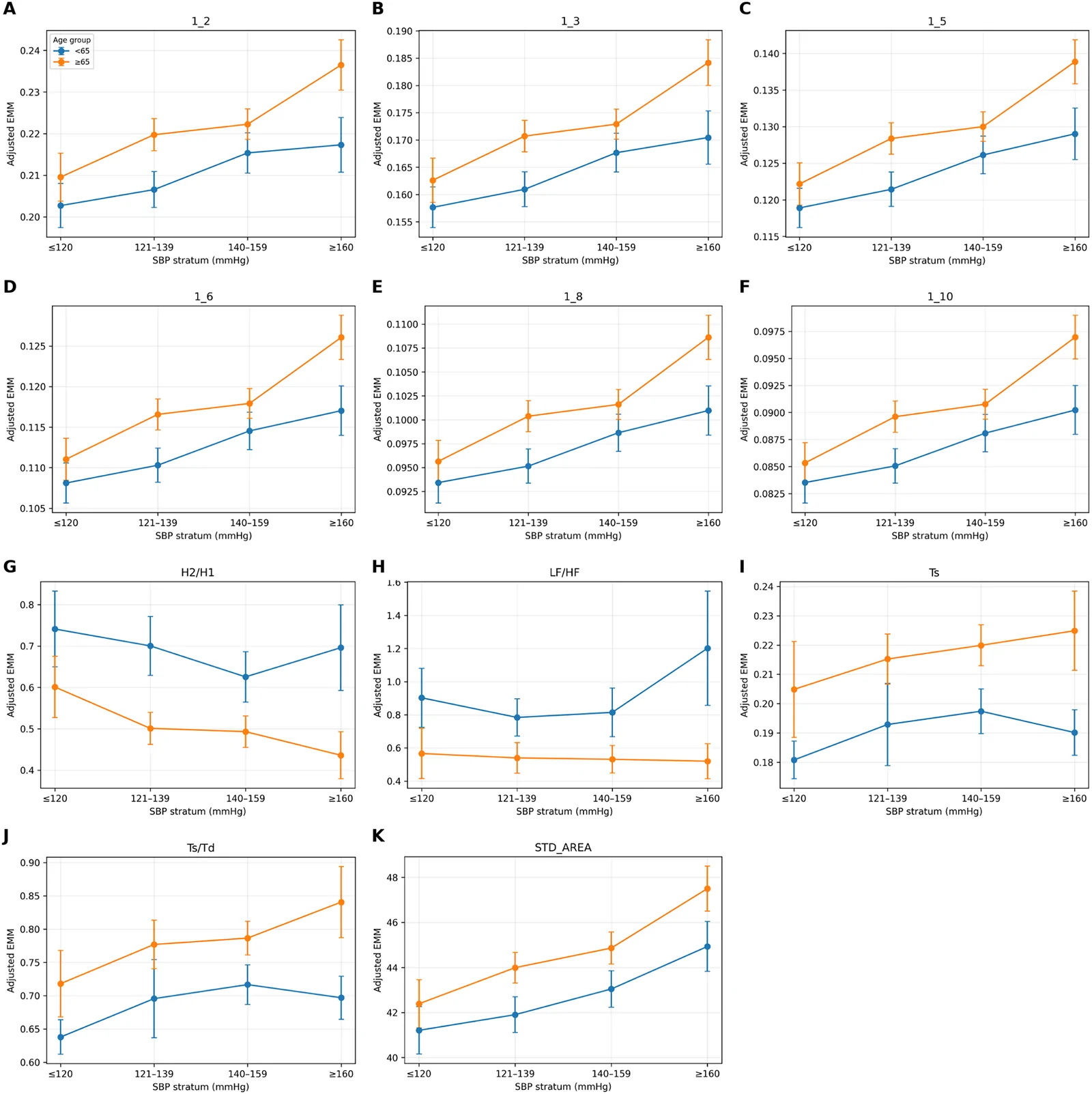
Covariate-adjusted estimated marginal means (EMMs) with model-based 95% confidence intervals for the 11 candidate photoplethysmography (PPG)-derived features according to age group (<65 vs. ≥65 years) and measured systolic blood pressure (SBP) stratum. Separate multivariable linear models were fitted for each feature, including age group, categorical SBP stratum, the age-group × SBP-stratum interaction, measured SBP, sex, body mass index, diabetes mellitus, coronary artery disease, congestive heart failure, chronic kidney disease, and antihypertensive medication use (calcium channel blockers, ACE inhibitors/angiotensin receptor blockers, β-blockers, α-blockers, and thiazide diuretics). Covariates other than age group and SBP stratum were fixed at their observed sample means. Error bars represent model-based 95% confidence intervals for the adjusted estimates. **(A–K)** represent 1_2, 1_3, 1_5, 1_6, 1_8, 1_10, H2/H1, LF/HF, Ts, Ts/Td, and STD_AREA, respectively. For Ts and Ts/Td, one participant with physiologically implausible negative timing values was excluded from the corresponding feature-specific analyses only.

To formally assess whether the association between age and each candidate PPG feature differed across measured SBP strata, additional models included an interaction between continuous age and categorical SBP stratum. Omnibus Wald tests were used to evaluate the joint significance of the age × SBP-stratum interaction terms. The resulting interaction P-values were adjusted across the 11 candidate features using the Benjamini–Hochberg false discovery rate procedure.

As a sensitivity analysis for potential non-linearity, a quadratic age term (age^2^) was additionally included in each fully adjusted model. Physiologically implausible negative values of systolic duration (Ts) and systolic-to-diastolic duration ratio (Ts/Td) were treated as invalid only for analyses involving the corresponding timing features. One recording yielded physiologically implausible negative Ts and Ts/Td values, most likely reflecting erroneous fiducial-point ordering or beat-level landmark pairing during automated timing extraction. Because the recording otherwise satisfied the predefined signal-quality criteria and independently derived non-timing features remained valid, only the affected timing variables were excluded for that participant. Continuous variables were standardized before model fitting, and standardized regression coefficients with heteroscedasticity-consistent type 3 (HC3) robust 95% confidence intervals were reported. The use of linear regression was based on examination of model residuals rather than on the marginal distribution of each PPG outcome. Residual and influence diagnostics, including tests for heteroscedasticity, residual normality, and influential observations, were examined for each model. Because some models showed departures from homoscedasticity or residual normality, HC3 robust standard errors were used for inference. Benjamini–Hochberg false discovery rate correction was applied separately to the age coefficients in the primary models and to the quadratic age terms in the sensitivity analyses across the 11 candidate features.

All data were analyzed using NumPy, SciPy, pandas, and statsmodels, and the results were organized into structured tables for subsequent interpretation.

## 3 Results

### 3.1 Baseline characteristics

**Table 1** lists participants’ baseline characteristics stratified by age (<65 vs. ≥65 years). This study included 1163 participants, with more males in the younger group than in the older group (64.02% vs. 49.70%, P< 0.001).

**Table 1:** Baseline characteristics of the study participants.

A. Baseline characteristics stratified by age group				
Characteristic		Age< 65 (n=503)	Age ≥ 65 (n=660)	P-value
Sex (male)		64.02%	49.70%	<0.001*
DM		26.69%	41.79%	<0.001*
Dyslipidemia		57.46%	65.28%	0.008*
CAD		30.48%	37.48%	0.015*
CHF		18.00%	12.94%	0.022*
COPD/Asthma		0.60%	2.73%	0.013*
CKD (eGFR< 60 mL/min/1.73 m^2^)		8.70%	27.62%	<0.001*
Medication				
CCB		43.95%	52.23%	0.007*
ACEI/ARB		55.85%	56.84%	0.784
β-blocker		44.56%	46.54%	0.542
α-blocker		3.43%	7.68%	0.004*
Thiazide		8.47%	9.83%	0.492

B. Age and sex distribution across measured SBP strata				
Characteristic	≤120 mmHg	121–139 mmHg	140–159 mmHg	≥160 mmHg
n	200	418	374	171
Age, years, mean ± SD	61.65 ± 14.38	64.84 ± 12.97	66.80 ± 11.62	67.22 ± 13.51
Male, n (%)	107 (53.5%)	236 (56.5%)	223 (59.6%)	84 (49.1%)

DM, diabetes mellitus; CAD, coronary artery disease; CHF, congestive heart failure; COPD, chronic obstructive pulmonary disease; CKD, chronic kidney disease; eGFR, estimated glomerular filtration rate; CCB, calcium channel blocker; ACEI, angiotensin converting enzyme inhibitor; ARB, angiotensin 2 receptor blocker.

*P < 0.05 indicates statistical significance.

The prevalence of cardiometabolic comorbidities differed significantly between age groups. Participants aged ≥65 years exhibited higher rates of DM (P< 0.001), dyslipidemia (P = 0.008), CAD (P = 0.015), chronic obstructive pulmonary disease or asthma (P = 0.013), and CKD (P< 0.001). Conversely, CHF was more prevalent in those aged<65 years (P = 0.022).

Regarding medication use, calcium channel blockers and α-blockers were more frequently prescribed in the older group than in the younger group (P = 0.007 and 0.004, respectively). Meanwhile, the use of angiotensin‐converting enzyme inhibitors, angiotensin receptor blockers, β-blockers, or thiazide diuretics showed no significant differences between such groups.

Given the study’s observational nature, several baseline characteristics differed between the two age groups. Therefore, these variables were considered potential confounders and adjusted for in subsequent multivariable analyses.

### 3.2 Age-related differences in PPG features within each SBP stratum

In total, 1163 participants were classified into four SBP strata, each comprising both younger (<65 years) and older (≥65 years) adults. **Table 2** summarizes the sample size distribution of each age group across the strata.

**Table 2:** Quantitative comparison of the 11 candidate age-associated PPG features across measured SBP strata.

SBP group (mmHg)	≤120		121–139		140–159		≥160	
Age group (years)	<65	≥65	<65	≥65	<65	≥65	<65	≥65
n	112	88	188	230	141	233	62	109
1_2	0.2019	0.2119	0.2056	0.2215	0.2118	0.2239	0.2135	0.2396
1_3	0.1568	0.1646	0.1603	0.1721	0.1651	0.1741	0.1673	0.1868
1_5	0.1180	0.1237	0.1209	0.1295	0.1243	0.1308	0.1265	0.1409
1_6	0.1073	0.1124	0.1099	0.1175	0.1129	0.1187	0.1148	0.1280
1_8	0.0927	0.0968	0.0948	0.1012	0.0973	0.1022	0.0991	0.1102
1_10	0.0829	0.0864	0.0847	0.0903	0.0869	0.0913	0.0886	0.0984
H2/H1	0.7319	0.5917	0.7391	0.4965	0.6657	0.4645	0.7107	0.4074
LF/HF	0.8526	0.5919	0.7872	0.5452	0.8510	0.5112	1.1227	0.4833
Ts	0.1852	0.2078	0.1923	0.2147	0.1910	0.2200	0.1889	0.2264
Ts/Td	0.6550	0.7338	0.6880	0.7779	0.6879	0.7878	0.6832	0.8532
STD_AREA	41.1850	42.9635	41.9495	44.1691	42.5847	44.8249	44.1992	47.8491

H1, harmonic 1; H2, harmonic 2; LF, low frequency; HF, high frequency; Ts/Td, systolic to diastolic duration ratio; STD_AREA, standard deviation of the pulse area.

For Ts and Ts/Td only, one physiologically implausible negative value was excluded from the ≥65-year subgroup in the ≤120-mmHg stratum, resulting in n = 87 for these two timing variables; all other features in this subgroup retained n = 88.

The values represent means for younger (<65 years) and older (≥65 years) adults within each SBP group.

In each SBP stratum, exploratory age-group differences in PPG features were first evaluated using the Mann–Whitney U test with a nominal two-sided P< 0.05 threshold. These nominally significant features were used for the subsequent intersection analysis. Multiplicity-adjusted results based on the Benjamini–Hochberg procedure were additionally examined as a sensitivity analysis. Complete Mann–Whitney U test results, FDR-adjusted q-values, and rank-biserial effect sizes for the 11 candidate features are provided in **Supplementary Table S3**. For Ts and Ts/Td, one participant in the ≥65-year subgroup with SBP ≤120 mmHg had physiologically implausible negative timing values and was excluded from analyses of these two variables only, yielding n = 87 for the corresponding timing comparisons. Other independently derived PPG features from this recording were retained because the recording satisfied the predefined signal-quality criteria. Corresponding complete screening results for all 27 PPG-derived features across all four measured SBP strata are provided in **Supplementary Table S4**.

#### 3.2.1 SBP ≤120 mmHg

In the SBP ≤120-mmHg group, several PPG-derived features significantly differed between the<65- and ≥65-year groups. Older adults had significantly higher normalized waveform width indices, including 1_10, 1_2, 1_3, 1_5, 1_6, and 1_8 (all P ≤ 0.001), and significantly lower harmonic ratios, specifically H2/H1 and H3/H1 (P = 0.040 and 0.035, respectively), than the younger ones. PRV-derived spectral indices also differed between the age groups. Older adults had modestly lower LF values (P = 0.007) and significantly lower LF/HF (P< 0.001) than the younger ones.

Amplitude-related variability was increased in the ≥65-year group, as indicated by the higher standard deviation of the pulse area (STD_AREA) (42.96 vs. 41.18, P = 0.002; rank-biserial r = 0.257). In the same age group, time-domain analysis revealed prolonged systolic duration (Ts) (P< 0.001) and higher Ts/Td ratios (P< 0.001) compared with those in the younger group.

#### 3.2.2 SBP = 121–139 mmHg

In the SBP 121–139-mmHg category, significant age-related differences were observed across multiple PPG-derived waveform features. All six normalized waveform width indices (1_10, 1_2, 1_3, 1_5, 1_6, and 1_8; all, P< 0.001) were significantly higher in older adults (≥65 years) than in the younger ones (<65 years); thus, the older group may have consistently greater normalized waveform widths at the predefined fractional systolic peak levels. In addition, the integrated pulse wave area, which represents the overall amplitude envelope per cardiac cycle, was higher (133.83 vs. 110.70, P = 0.010), while BMI was significantly lower in the older group than in the younger group (P< 0.001).

Harmonic indices also significantly differed between such age groups. H2/H1 was significantly lower in older adults (P< 0.001), reflecting lower relative higher-harmonic magnitude than the fundamental frequency. PRV-derived spectral indices exhibited a mixed pattern. In particular, the older group had higher LF values (P = 0.005) but exhibited significantly lower LF/HF ratios (P< 0.001) than the younger group. Additionally, beat-to-beat variability parameters, including pNN50 and RMSSD, were higher in older adults (both, P< 0.001), reflecting wider variability in pulse intervals. STD_AREA, which represents pulse amplitude variability, was also significantly higher among older adults (P< 0.001).

Changes were observed in time-domain waveform structure. Older adults had slightly shorter diastolic duration (Td) (P = 0.034) and markedly prolonged Ts (P< 0.001). Furthermore, the older group had significantly higher Ts/Td ratio (P< 0.001) and peak-to-peak interval (ΔT) (P = 0.003).

#### 3.2.3 SBP = 140–159 mmHg

In the SBP 140–159-mmHg category, older adults exhibited consistently higher values across all six normalized waveform width indices than the younger adults (all, P< 0.001). The integrated pulse wave area, which represents the overall amplitude envelope per cardiac cycle, was also markedly higher in older adults (136.06 vs. 115.73, P = 0.001). Contrarily, BMI was lower in this age group (P< 0.001).

Harmonic and PRV-derived spectral indices demonstrated clear age-related differences. The older group had markedly lower H2/H1 and LF/HF ratios than the younger group (P< 0.001 and P< 0.001, respectively). Several rhythm- and variability-related features, including pNN50 and RMSSD, were also elevated among older adults (both, P< 0.001). Additionally, the older group had significantly higher STD_AREA (P< 0.001) and exhibited a modest but statistically significant increase in SDNN (P = 0.040).

Time-domain intervals displayed consistent patterns with age. The older group had substantially longer Ts, higher Ts/Td ratio, and greater ΔT than the younger group (all, P< 0.001).

#### 3.2.4 SBP ≥160 mmHg

In the SBP ≥160-mmHg group, older adults exhibited significantly higher values across all six normalized waveform width indices than younger adults (all, P< 0.001). This age group had a significantly greater integrated pulse wave area (167.18 vs. 126.66, P = 0.028) and a substantially lower BMI (P< 0.001).

Harmonic indices exhibited pronounced age-related differences. Both the H2/H1 and H3/H1 ratios significantly decreased in older adults (P< 0.001 and P = 0.001, respectively). PRV-derived spectral indices demonstrated divergent changes; specifically, the HF values were higher (P< 0.001), whereas the LF/HF ratios were markedly lower (P< 0.001) in the older group. Moreover, measures of beat-to-beat interval variability, including pNN50 and RMSSD, were markedly elevated in older participants (both, P< 0.001). Pulse amplitude variability, measured using STD_AREA, and SDNN were also significantly higher in the older group (both, P< 0.001).

Time-domain waveform metrics showed consistent patterns with those observed in lower SBP categories. Older adults had shorter Td, prolonged Ts, and higher Ts/Td ratios than the younger ones (all, P< 0.001). In addition, ΔT was increased in this age group (P< 0.001).

### 3.3 Intersection of age-associated PPG features across SBP strata

**Figure 3** presents the UpSet plot summarizing the overlap of age-associated PPG features across SBP strata (≤120, 121–139, 140–159, and ≥160 mmHg). Using the nominal P< 0.05 threshold for the exploratory intersection analysis, the largest intersection comprised 11 candidate features showing age-group differences across all four measured SBP strata. After Benjamini–Hochberg correction across all 27 screened features within each stratum, 10 of the 11 candidate features remained significant across all four strata. H2/H1 was the only exception in the ≤120-mmHg stratum (P = 0.040; q = 0.077).

**Figure 3:**
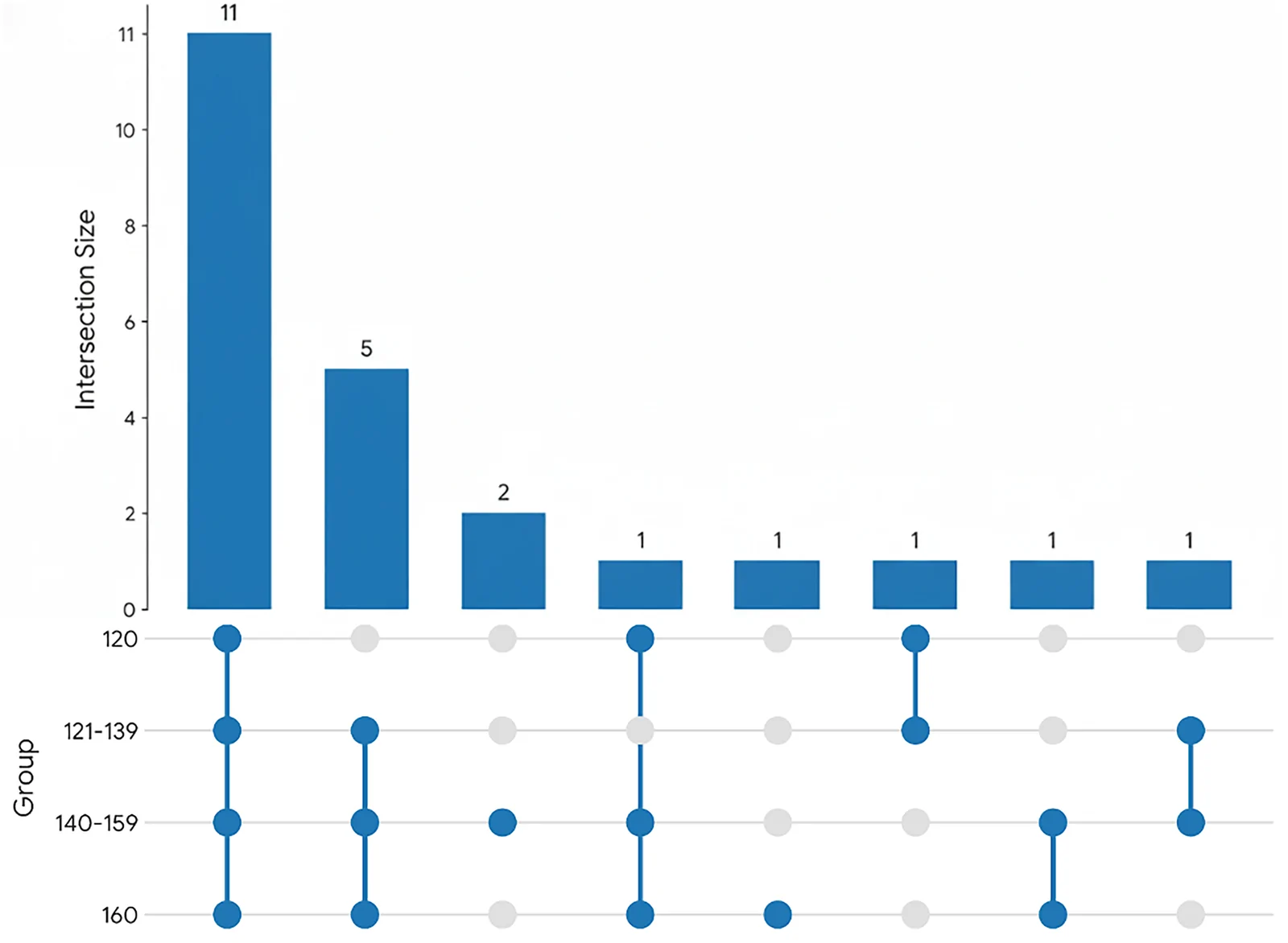
UpSet plot showing the overlap of PPG features with nominally significant age-group differences (P< 0.05 by Mann–Whitney U test) across the four measured SBP strata. The intersections represent descriptive overlap of features meeting the nominal significance criterion within each stratum and should not be interpreted as a formal statistical test of effect consistency across SBP strata. Formal effect modification was assessed separately using age × SBP-stratum interaction models.

These 11 candidate shared features were as follows:

Normalized waveform width indices: 1_2, 1_3, 1_5, 1_6, 1_8, and 1_10;

Morphological timing parameters: Ts and Ts/Td;

Waveform amplitude/area parameter: STD_AREA; and

Harmonic and PRV-derived spectral indicators: H2/H1 and LF/HF.

In all SBP categories, older adults consistently showed higher values for all six normalized waveform width indices, Ts, Ts/Td, and STD_AREA, alongside lower H2/H1 and LF/HF values. These directional patterns were consistent across the four measured SBP strata; thus, these 11 features may represent candidate age-associated PPG characteristics observed across the SBP spectrum. Their associations with continuous age were further evaluated in subsequent multivariable analyses.

The largest intersection comprised 11 candidate features shared across all four measured SBP strata. Smaller intersections represented features shared across subsets of two or three strata.

Collectively, although the number of statistically significant age group differences varied among the SBP strata, this exploratory intersection analysis identified 11 candidate age-associated PPG features showing nominally significant differences across all four strata. Furthermore, formal age × SBP-stratum interaction testing showed no statistically detectable effect modification after FDR correction; however, this finding should not be interpreted as evidence of equivalence of age effects across SBP strata.

### 3.4 Quantitative comparison of the 11 age-associated features across SBP strata

**Table 2** summarizes the unadjusted mean values of the 11 candidate PPG features for younger and older adults within each measured SBP stratum. These features, as mentioned above, included six normalized waveform width indices (1_2, 1_3, 1_5, 1_6, 1_8, and 1_10), two timing parameters (Ts and Ts/Td), one pulse area variability parameter (STD_AREA), the harmonic ratio H2/H1, and the PRV-derived spectral index LF/HF.

**Figure 2** presents covariate-adjusted estimated marginal means (EMMs) with 95% confidence intervals for the 11 candidate PPG features according to age group and measured SBP stratum. The adjusted estimates were derived from models including age group, SBP stratum, the age-group × SBP-stratum interaction, measured SBP, sex, BMI, DM, CAD, CHF, CKD, and antihypertensive medication use. After covariate adjustment, the overall directional patterns remained similar to the unadjusted comparisons, with older adults generally showing higher values for the six normalized waveform width indices, Ts, Ts/Td, and STD_AREA, and lower H2/H1 and LF/HF values across measured SBP strata.

Unlike the unadjusted descriptive means presented in **Table 2**, **Figure 2** displays covariate-adjusted estimates obtained from the multivariable models, with error bars representing model-based 95% confidence intervals. The plotted estimates were regenerated from the original participant-level dataset rather than derived from the previously presented unadjusted group means.

### 3.5 Adjusted associations between age and the 11 candidate PPG features

In multivariable linear regression analyses, all 11 candidate PPG features remained significantly associated with continuous age after adjustment for measured SBP, sex, BMI, DM, CAD, CHF, CKD, and antihypertensive medication use. Increasing age was positively associated with all six normalized waveform width indices, Ts, Ts/Td, and STD_AREA, whereas H2/H1 and LF/HF were negatively associated with age. The positive standardized age coefficients ranged from 0.186 to 0.229, while H2/H1 (β = −0.278, 95% CI −0.357 to −0.199) and LF/HF (β = −0.245, 95% CI −0.311 to −0.179) showed inverse associations. All 11 associations remained significant after Benjamini–Hochberg FDR correction (**Table 3**). Model explanatory power varied across outcomes, with adjusted R^2^ values ranging from 0.066 to 0.204. Maximum VIF values were below 3 for all models, providing no evidence of problematic multicollinearity. Full residual and influence diagnostics are provided in **Supplementary Table S1**. Although residual normality was not satisfied for several outcomes and heteroscedasticity was detected in a limited number of models, the HC3 robust inference was retained to reduce sensitivity to these assumption violations. Additional sensitivity analyses incorporating a quadratic age term showed no evidence of substantial non-linearity for any of the 11 candidate PPG features after FDR correction (all q = 0.993).

**Table 3:** Multivariable associations between continuous age and the 11 candidate PPG features.

Feature	Standardized β for age	95% CI	P-value	FDR-adjusted P-value	R^2^	Adjusted R^2^
1_2	0.212	0.145–0.278	4.01 × 10^-10^	4.41 × 10^-10^	0.168	0.158
1_3	0.226	0.165–0.287	4.43 × 10^-13^	6.08 × 10^-13^	0.195	0.185
1_5	0.228	0.168–0.288	1.12 × 10^-13^	2.05 × 10^-13^	0.209	0.199
1_6	0.228	0.168–0.288	9.59 × 10^-14^	2.05 × 10^-13^	0.209	0.200
1_8	0.229	0.169–0.289	6.83 × 10^-14^	2.05 × 10^-13^	0.212	0.202
1_10	0.228	0.168–0.287	9.21 × 10^-14^	2.05 × 10^-13^	0.213	0.204
H2/H1	−0.278	−0.357 to −0.199	4.48 × 10^-12^	5.47 × 10^-^¹^2^	0.170	0.160
LF/HF	−0.245	−0.311 to −0.179	2.55 × 10^-13^	4.01 × 10^-13^	0.085	0.074
Ts	0.201	0.156–0.246	2.48 × 10^-18^	2.73 × 10^-17^	0.077	0.066
Ts/Td	0.186	0.143–0.230	3.15 × 10^-17^	1.73 × 10^-16^	0.085	0.074
STD_AREA	0.207	0.142–0.272	5.54 × 10^-10^	5.54 × 10^-10^	0.176	0.166

Separate multivariable linear regression models were constructed for each PPG feature. Age was modeled as a continuous independent variable. Models were adjusted for measured SBP, sex, BMI, DM, CAD, CHF, CKD, and antihypertensive medication use, including calcium channel blockers, ACE inhibitors/angiotensin receptor blockers, β-blockers, α-blockers, and thiazide diuretics. Continuous variables were standardized before model fitting. Standardized regression coefficients for age and heteroscedasticity-consistent type 3 (HC3) robust 95% confidence intervals are presented. P-values for the age coefficients were adjusted across the 11 models using the Benjamini–Hochberg false discovery rate procedure. Physiologically implausible negative Ts and Ts/Td values were excluded only from analyses involving the corresponding timing feature. CI, confidence interval; FDR, false discovery rate; BMI, body mass index; DM, diabetes mellitus; CAD, coronary artery disease; CHF, congestive heart failure; CKD, chronic kidney disease.

### 3.6 Interaction between age and SBP stratum

Formal age × SBP-stratum interaction analyses showed no significant effect modification for any of the 11 candidate PPG features after FDR correction (all q ≥ 0.350; **Supplementary Table S2**). These findings provided no statistical evidence that the continuous-age associations differed substantially across measured SBP strata.

## 4 Discussion

The descriptive SBP-stratified analyses identified 11 candidate PPG features that differed between adults aged<65 and ≥65 years across all four measured SBP strata. Importantly, the primary inferential analyses treated age as a continuous variable rather than a dichotomous exposure. All 11 candidate features remained significantly associated with continuous age after adjustment for measured SBP, sex, BMI, DM, CAD, CHF, CKD, and antihypertensive medication use. Additional quadratic-age sensitivity analyses showed no evidence of substantial non-linearity after FDR correction.

The exploratory intersection analysis identified 11 candidate features using nominal within-stratum significance. In the multiplicity-adjusted sensitivity analysis, 10 of these features retained significance across all four SBP strata, whereas H2/H1 did not meet the FDR-adjusted threshold in the ≤120-mmHg group. Nevertheless, H2/H1 remained strongly associated with continuous age in the fully adjusted model.

The identified features encompassed waveform geometry, harmonic composition, pulse rate variability, systolic timing, and pulse area variability; thus, age-associated changes in peripheral PPG signals may involve multiple physiological domains. Nevertheless, given that this research is a cross-sectional observational study and that established vascular measurements, including PWV and central blood pressure, were not obtained, these findings should be interpreted not as validated biomarkers or direct measures of vascular aging but as robust age-associated PPG characteristics.

Across all SBP categories, older adults exhibited higher values in all six normalized waveform width indices. These indices quantify the horizontal width of the systolic waveform at predefined fractional amplitudes, normalized to beat duration. Accordingly, the observed increases indicate broader normalized systolic waveform contours rather than increased “sharpness” in the conventional geometric sense. This direction is consistent with the numerical findings of our previous study, in which these indices also increased across higher measured SBP categories (Lin et al., 2025). The earlier statement in the Abstract of that publication describing a decline in these indices was a directional wording error and did not reflect the reported numerical results. Age-related changes in arterial wall properties and pulse-wave transmission may contribute to these alterations in peripheral waveform geometry. However, because arterial stiffness and wave-reflection parameters were not directly measured in the present study, these indices should not be interpreted as direct measures of arterial stiffness (Millasseau et al., 2002).

Earlier return of reflected waves may additionally modify systolic waveform configuration and late systolic loading (McEniery et al., 2005). However, these findings should not be interpreted as direct evidence of arterial stiffening because PWV and wave reflection parameters were not directly measured in the present study. Further validation against established vascular measurements is required before considering these indices as vascular aging biomarkers (Mattace-Raso et al., 2006).

The H2/H1 ratio was consistently lower in older adults across all SBP categories. H2 captures the contribution of the arterial tree’s reflective and resonant behavior, whereas H1 represents the cardiac cycle’s fundamental frequency (Park et al., 2023). A lower H2/H1 ratio indicates reduced harmonic complexity and altered propagation of HF waveform components (O'Rourke and Hashimoto, 2007). However, given that arterial stiffness was not directly measured, this interpretation remains inferential.

Younger adults have elastic vessels that support complex oscillatory patterns, enabling pulsatile energy to be stored and released with high fidelity. However, when arterial compliance decreases with aging, pulse wave transmission can be altered, resulting in a waveform that is increasingly dominated by the fundamental systolic component. These findings reflect age-associated alterations in harmonic waveform composition, especially among participants in the lowest measured SBP stratum (≤120 mmHg) (Goldberger et al., 2002).

The LF/HF ratio exhibited a clear and consistent reduction with aging across all measured SBP strata. Age-related changes in heart-rate variability and autonomic regulation have been reported previously (Voss et al., 2015) and aging has also been associated with altered baroreflex function (Monahan, 2007).

These mechanisms may contribute, at least in part, to the observed differences in LF/HF. Accordingly, the observed age-associated reduction in LF/HF should not be interpreted as direct evidence of altered sympathetic–parasympathetic balance, but rather as a PRV-derived spectral characteristic influenced by multiple cardiovascular and peripheral factors (Billman, 2013). The absence of a significant LF/HF difference across SBP categories in our previous study does not conflict with the present findings, because the previous analysis addressed variation across measured SBP levels, whereas the current analysis specifically examines age-associated differences within SBP strata. More broadly, age-related reductions in physiological adaptability have been noted across multiple regulatory systems (Lipsitz, 2002).

Across all measured SBP strata, Ts and the Ts/Td ratio increased consistently with age. Therefore, alterations in the temporal configuration of the peripheral PPG waveform appear to be associated with age. Age-related changes in arterial compliance and pulse wave transmission may influence peripheral pulse timing (Lakatta, 2003).

However, in this study, Ts and Ts/Td were derived from the finger PPG waveform rather than from direct measurements of ventricular ejection time. Although systolic time intervals are proven physiologically relevant in cardiovascular assessment (Boudoulas, 1990), the present PPG-derived parameters should not be interpreted as direct measures of ventricular systolic duration. Similarly, given that ventricular–arterial coupling was not directly measured, any insights involving ventricular and vascular stiffening should still be interpreted with caution (Faconti et al., 2015).

STD_AREA, which represents beat-to-beat variability in a pulse wave area, was consistently higher in older adults than in younger adults across all SBP strata, indicating greater variability in peripheral pulse wave characteristics with increasing age. Age-related alterations in arterial properties and peripheral pulse transmission may contribute to pulse wave behavior changes (O'Rourke and Hashimoto, 2007).

However, the physiological determinants of STD_AREA were not directly assessed in this study. Therefore, higher STD_AREA should be interpreted not as a direct measure of pulsatile energy, arterial stiffness, or microvascular injury but as an age-associated PPG characteristic. Validation against established central and peripheral vascular measurements remains necessary (O'Rourke and Safar, 2005).

The 11 age-associated PPG features identified across all measured SBP strata may have potential clinical relevance. First, these findings suggest that brachial SBP alone cannot fully capture age-related differences in peripheral pulse wave characteristics. Older adults with measured SBP ≤120 mmHg (the lowest stratum) still exhibited distinct PPG patterns, including higher normalized waveform width indices, prolonged systolic timing, lower H2/H1 and LF/HF ratios, and greater pulse area variability. Therefore, PPG may provide complementary information on age-associated vascular and PRV-derived spectral characteristics beyond a single brachial SBP measurement. However, the clinical and prognostic significance of these features still needs to be further validated against established vascular measurements and cardiovascular outcomes.

Second, features such as normalized waveform width indices, Ts/Td, and STD_AREA could be potential noninvasive indicators of age-associated changes in peripheral pulse wave characteristics. Given that their potential relevance to microvascular stress remains speculative, validation against established vascular measurements and clinical outcomes is warranted.

Third, the LF/HF ratio observed in older adults was consistently lower, suggesting age-associated differences in PRV-derived spectral characteristics. However, this study did not directly assess arrhythmia risk, orthostatic responses, or chronotropic competence. Therefore, the clinical implications of this finding should be interpreted cautiously.

Collectively, PPG-derived features may provide complementary information regarding age-associated peripheral pulse wave characteristics beyond a single brachial SBP measurement. Given that such features consistently demonstrated age group differences in participants with measured SBP ≤120 mmHg, similar brachial SBP values do not necessarily mean identical peripheral waveform characteristics. However, this study did not directly measure central arterial stiffness, wave reflection, or ventricular–arterial coupling. Hence, the physiological and clinical implications of these differences require validation using established vascular measurements and prospective cardiovascular outcomes (Kaess et al., 2012).

### 4.1 Limitations

Our study has several limitations that should be acknowledged. First, the cross-sectional design precludes causal inference and does not allow assessment of longitudinal changes in blood pressure, PPG characteristics, or cardiovascular outcomes. Second, although antihypertensive medication classes were included as covariates in the revised multivariable models, residual medication-related confounding cannot be completely excluded because medication dose, treatment duration, adherence, and combination therapy were not modeled. Antihypertensive agents may influence peripheral vascular tone and PPG waveform morphology; therefore, future studies with more detailed pharmacological information are warranted. Third, central blood pressure and pulse wave velocity (PWV), established reference measures of central hemodynamics and arterial stiffness, were not obtained; consequently, the proposed physiological interpretations could not be directly validated.

Because LF/HF was derived from 90-s recordings, it should be interpreted as a short-recording PRV-derived spectral index rather than as a conventional long-duration HRV measure, and the precision of its low-frequency component may be limited.

Fourth, peripheral PPG measurements may be influenced by local temperature, peripheral perfusion, and other acquisition-related factors despite the application of signal-quality control procedures. Finally, this was a single-center study involving cardiology outpatients, which may limit generalizability to community-based populations and other ethnic or clinical groups. External validation in larger, multicenter cohorts is therefore warranted.

In addition, candidate-feature selection and subsequent multivariable regression were performed in the same dataset; therefore, the regression analyses do not constitute independent validation. Independent external validation remains necessary.

PPG-based assessment may ultimately serve as a simple and noninvasive adjunct to conventional cardiovascular evaluation. However, this study did not examine clinical risk reclassification, cost-effectiveness, treatment decision making, or direct measures of arterial stiffness. Therefore, before these PPG-derived features can be applied to individualized vascular-risk assessment, prospective studies incorporating established vascular measurements and cardiovascular outcomes are required.

Longitudinal studies are also needed to determine whether normalized waveform width indices, Ts/Td, harmonic alterations, or pulse area variability can predict transitions to systolic hypertension, HFpEF, CKD, or cognitive decline. Additionally, mechanistic studies including central hemodynamic measurements (e.g., augmentation index and carotid–femoral PWV) would aid in validating the physiological interpretations proposed in this study.

By applying machine learning models that integrate multiple PPG features, vascular aging profiles may be individualized to early detect diseases or monitor treatment. In addition, emerging wearable technologies may allow continuous tracking of vascular aging in real-world settings, enabling clinicians to potentially detect dynamic changes that are missed by clinic measurements. Finally, interventional trials evaluating exercise, diet, medications, or device-based therapies on these PPG features may identify modifiable pathways for vascular improvement.

## 5 Conclusions

This study identified 11 candidate PPG-derived features showing nominally significant age-group differences across all four measured SBP strata, of which 10 retained significance across all four strata after within-stratum FDR correction. Older age was associated with higher values of six normalized waveform width indices, Ts, Ts/Td, and STD_AREA, alongside lower H2/H1 and LF/HF ratios. These directional patterns were observed across the full measured SBP spectrum, especially among adults with measured SBP ≤ 120 mmHg. After adjustment for measured SBP, sex, BMI, DM, CAD, CHF, CKD, and antihypertensive medication use, all 11 features remained significantly associated with continuous age.

Therefore, aging may be associated with coordinated changes in peripheral PPG waveform geometry, pulse timing, harmonic composition, pulse area variability, and PRV-derived spectral characteristics. Nevertheless, the identified features should currently be considered not as validated measures of arterial stiffness or cardiovascular risk but as candidate age-associated PPG characteristics. Longitudinal and multicenter studies incorporating PWV, central blood pressure, medication effects, peripheral temperature, and cardiovascular outcomes are required to clarify their physiological significance and potential clinical applicability. Once validated, these features may facilitate in developing interpretable, sensor-based approaches for the noninvasive assessment of cardiovascular aging.

## Data Availability

The data generated or analyzed during the current study are not publicly available because they contain sensitive personal health information collected from patients, and public disclosure could compromise confidentiality. The data were collected and analyzed under approval from the Research Ethics Committee of China Medical University and Hospital, with informed consent obtained from the study participants. De-identified data may be made available upon reasonable request to the corresponding author, subject to the approval of the relevant ethics review board and applicable institutional requirements. Requests should be directed to Po-Yen Ko (013402@tool.caaumed.org.tw).

## Supplementary material

Supplementary Table 1 Regression model diagnostics for the 11 candidate PPG features. VIF, variance inflation factor. The maximum VIF represents the largest VIF observed among predictors included in each model. Breusch–Pagan tests were used to assess heteroscedasticity, Jarque–Bera tests to assess residual normality, and Cook’s distance to characterize potentially influential observations. Maximum VIF values were<3 in all models, indicating no evidence of problematic multicollinearity. Because residual non-normality and heteroscedasticity were present for some outcomes, HC3 heteroscedasticity-robust standard errors and confidence intervals were used for statistical inference.

Supplementary Table 2 Omnibus interaction tests between continuous age and SBP stratum for the 11 candidate PPG features. Omnibus Wald tests were used to assess the joint significance of the age × SBP-stratum interaction terms. Models were adjusted for sex, BMI, DM, CAD, CHF, CKD, and antihypertensive medication use. P-values were adjusted across the 11 candidate features using the Benjamini–Hochberg false discovery rate procedure.

Supplementary Table 3 Mann–Whitney U comparisons and effect sizes for the 11 candidate PPG features across SBP strata. Values are presented as mean ± standard deviation. Between-group comparisons were performed using the Mann–Whitney U test. Rank-biserial correlation was used as the corresponding effect-size measure; positive values indicate higher feature values in participants aged ≥65 years, whereas negative values indicate lower values in the ≥65-year group. Benjamini–Hochberg false discovery rate correction was applied separately within each SBP stratum across all 27 PPG features included in the initial screening analysis. Physiologically implausible negative Ts and Ts/Td values were treated as invalid for analyses involving the corresponding timing features, resulting in n = 87 for the ≥65-year subgroup in the ≤120-mmHg stratum for these two variables. FDR, false discovery rate; LF, low frequency; HF, high frequency; Ts/Td, systolic-to-diastolic duration ratio; STD_AREA, standard deviation of beat-wise pulse area.

Supplementary Table 4 Complete Mann–Whitney U screening results for all 27 PPG-derived features across measured SBP strata.
