# Supplementary material for: Age-associated photoplethysmographic features across systolic blood pressure strata: a cross-sectional study
Source: Front Physiol. 2026 Oct 29;17:1992759. doi: 10.3389/fphys.2026.1992759 (PMC13754651; doi:10.3389/fphys.2026.1992759)
Supplement: Supplementary file 1 [file Table1.docx]

**Supplementary Table S1. Regression model diagnostics for the 11 candidate PPG features**

| **Feature** | **Maximum VIF** | **Breusch–Pagan P-value** | **Jarque–Bera P-value** | **Maximum Cook’s D** |
| --- | --- | --- | --- | --- |
| 1_2 | 2.839 | 0.450 | <0.001 | 0.074 |
| 1_3 | 2.839 | 0.757 | <0.001 | 0.031 |
| 1_5 | 2.839 | 0.423 | <0.001 | 0.027 |
| 1_6 | 2.839 | 0.262 | <0.001 | 0.029 |
| 1_8 | 2.839 | 0.145 | <0.001 | 0.026 |
| 1_10 | 2.839 | 0.095 | <0.001 | 0.026 |
| H2/H1 | 2.839 | <0.001 | <0.001 | 0.058 |
| LF/HF | 2.839 | 0.011 | <0.001 | 0.070 |
| Ts | 2.845 | 0.088 | <0.001 | 0.332 |
| Ts/Td | 2.845 | 0.069 | <0.001 | 0.368 |
| STD_AREA | 2.839 | 0.789 | 0.092 | 0.036 |

VIF, variance inflation factor. The maximum VIF represents the largest VIF observed among predictors included in each model. Breusch–Pagan tests were used to assess heteroscedasticity, Jarque–Bera tests to assess residual normality, and Cook’s distance to characterize potentially influential observations. Maximum VIF values were <3 in all models, indicating no evidence of problematic multicollinearity. Because residual non-normality and heteroscedasticity were present for some outcomes, HC3 heteroscedasticity-robust standard errors and confidence intervals were used for statistical inference.
