# Supplementary material for: Age-associated photoplethysmographic features across systolic blood pressure strata: a cross-sectional study
Source: Front Physiol. 2026 Oct 29;17:1992759. doi: 10.3389/fphys.2026.1992759 (PMC13754651; doi:10.3389/fphys.2026.1992759)
Supplement: Supplementary file 2 [file Table2.docx]

Supplementary Table S2. Omnibus interaction tests between continuous age and SBP stratum for the 11 candidate PPG features

| **Feature** | **Omnibus P-value** | **FDR-adjusted q-value** |
| --- | --- | --- |
| 1_2 | 0.129 | 0.350 |
| 1_3 | 0.174 | 0.350 |
| 1_5 | 0.205 | 0.350 |
| 1_6 | 0.194 | 0.350 |
| 1_8 | 0.215 | 0.350 |
| 1_10 | 0.223 | 0.350 |
| H2/H1 | 0.651 | 0.695 |
| LF/HF | 0.096 | 0.350 |
| Ts | 0.654 | 0.695 |
| Ts/Td | 0.339 | 0.466 |
| STD_AREA | 0.695 | 0.695 |

Omnibus Wald tests were used to assess the joint significance of the age × SBP-stratum interaction terms. Models were adjusted for sex, BMI, DM, CAD, CHF, CKD, and antihypertensive medication use. P-values were adjusted across the 11 candidate features using the Benjamini–Hochberg false discovery rate procedure.
