# Supplementary material for: Age-associated photoplethysmographic features across systolic blood pressure strata: a cross-sectional study
Source: Front Physiol. 2026 Oct 29;17:1992759. doi: 10.3389/fphys.2026.1992759 (PMC13754651; doi:10.3389/fphys.2026.1992759)
Supplement: Supplementary file 3 [file Table3.docx]

Supplementary Table S3. Mann–Whitney U comparisons and effect sizes for the 11 candidate PPG features across SBP strata

| **SBP stratum** | **Feature** | **<65, mean ± SD** | **≥65, mean ± SD** | **P-value** | **FDR-adjusted q-value** | **Rank-biserial r** |
| --- | --- | --- | --- | --- | --- | --- |
| ≤120 | 1_2 | 0.2019 ± 0.0251 | 0.2119 ± 0.0262 | 0.00275 | 0.00743 | 0.247 |
| ≤120 | 1_3 | 0.1568 ± 0.0177 | 0.1646 ± 0.0184 | 0.000777 | 0.00372 | 0.277 |
| ≤120 | 1_5 | 0.1180 ± 0.0128 | 0.1237 ± 0.0132 | 0.000827 | 0.00372 | 0.276 |
| ≤120 | 1_6 | 0.1073 ± 0.0118 | 0.1124 ± 0.0118 | 0.000791 | 0.00372 | 0.277 |
| ≤120 | 1_8 | 0.0927 ± 0.0102 | 0.0968 ± 0.0101 | 0.00119 | 0.00461 | 0.267 |
| ≤120 | 1_10 | 0.0829 ± 0.0090 | 0.0864 ± 0.0086 | 0.00152 | 0.00512 | 0.262 |
| ≤120 | H2/H1 | 0.7319 ± 0.4522 | 0.5917 ± 0.3466 | 0.03999 | 0.07712 | −0.169 |
| ≤120 | LF/HF | 0.8526 ± 0.8469 | 0.5919 ± 0.7204 | 0.000494 | 0.00372 | −0.287 |
| ≤120 | Ts | 0.1852 ± 0.0248 | 0.2078 ± 0.0758 | 0.000127 | 0.00342 | 0.317 |
| ≤120 | Ts/Td | 0.6550 ± 0.1018 | 0.7338 ± 0.2226 | 0.000515 | 0.00372 | 0.287 |
| ≤120 | STD_AREA | 41.1850 ± 5.1256 | 42.9635 ± 4.9725 | 0.00181 | 0.00543 | 0.257 |
| 121–139 | 1_2 | 0.2056 ± 0.0289 | 0.2215 ± 0.0294 | 3.56 × 10⁻⁹ | 3.21 × 10⁻⁸ | 0.336 |
| 121–139 | 1_3 | 0.1603 ± 0.0214 | 0.1721 ± 0.0223 | 1.22 × 10⁻⁸ | 8.22 × 10⁻⁸ | 0.324 |
| 121–139 | 1_5 | 0.1209 ± 0.0156 | 0.1295 ± 0.0166 | 6.93 × 10⁻⁸ | 2.69 × 10⁻⁷ | 0.307 |
| 121–139 | 1_6 | 0.1098 ± 0.0140 | 0.1175 ± 0.0149 | 6.96 × 10⁻⁸ | 2.69 × 10⁻⁷ | 0.306 |
| 121–139 | 1_8 | 0.0948 ± 0.0118 | 0.1012 ± 0.0126 | 1.85 × 10⁻⁷ | 5.90 × 10⁻⁷ | 0.296 |
| 121–139 | 1_10 | 0.0847 ± 0.0105 | 0.0903 ± 0.0112 | 1.97 × 10⁻⁷ | 5.90 × 10⁻⁷ | 0.296 |
| 121–139 | H2/H1 | 0.7391 ± 0.4907 | 0.4965 ± 0.2923 | 5.25 × 10⁻⁸ | 2.69 × 10⁻⁷ | −0.309 |
| 121–139 | LF/HF | 0.7872 ± 0.7382 | 0.5452 ± 0.6993 | 1.68 × 10⁻⁶ | 4.54 × 10⁻⁶ | −0.272 |
| 121–139 | Ts | 0.1923 ± 0.0865 | 0.2147 ± 0.0637 | 6.02 × 10⁻¹⁴ | 1.62 × 10⁻¹² | 0.427 |
| 121–139 | Ts/Td | 0.6880 ± 0.3637 | 0.7779 ± 0.2714 | 1.94 × 10⁻¹² | 2.62 × 10⁻¹¹ | 0.400 |
| 121–139 | STD_AREA | 41.9495 ± 5.0815 | 44.1690 ± 5.1767 | 9.61 × 10⁻⁵ | 2.24 × 10⁻⁴ | 0.222 |
| 140–159 | 1_2 | 0.2118 ± 0.0273 | 0.2239 ± 0.0282 | 2.36 × 10⁻⁵ | 7.07 × 10⁻⁵ | 0.261 |
| 140–159 | 1_3 | 0.1651 ± 0.0203 | 0.1741 ± 0.0212 | 3.02 × 10⁻⁵ | 8.16 × 10⁻⁵ | 0.257 |
| 140–159 | 1_5 | 0.1243 ± 0.0147 | 0.1308 ± 0.0158 | 4.30 × 10⁻⁵ | 1.06 × 10⁻⁴ | 0.252 |
| 140–159 | 1_6 | 0.1129 ± 0.0132 | 0.1187 ± 0.0142 | 7.09 × 10⁻⁵ | 1.37 × 10⁻⁴ | 0.245 |
| 140–159 | 1_8 | 0.0973 ± 0.0111 | 0.1022 ± 0.0122 | 7.99 × 10⁻⁵ | 1.44 × 10⁻⁴ | 0.243 |
| 140–159 | 1_10 | 0.0869 ± 0.0098 | 0.0913 ± 0.0108 | 6.52 × 10⁻⁵ | 1.35 × 10⁻⁴ | 0.246 |
| 140–159 | H2/H1 | 0.6657 ± 0.3701 | 0.4645 ± 0.2760 | 1.22 × 10⁻⁸ | 1.10 × 10⁻⁷ | −0.351 |
| 140–159 | LF/HF | 0.8510 ± 0.8085 | 0.5112 ± 0.5799 | 1.16 × 10⁻⁷ | 6.26 × 10⁻⁷ | −0.327 |
| 140–159 | Ts | 0.1910 ± 0.0383 | 0.2200 ± 0.0490 | 1.69 × 10⁻¹⁴ | 4.57 × 10⁻¹³ | 0.473 |
| 140–159 | Ts/Td | 0.6879 ± 0.1494 | 0.7878 ± 0.1839 | 1.86 × 10⁻¹⁰ | 2.51 × 10⁻⁹ | 0.393 |
| 140–159 | STD_AREA | 42.5847 ± 4.5096 | 44.8249 ± 5.1991 | 5.70 × 10⁻⁵ | 1.28 × 10⁻⁴ | 0.248 |
| ≥160 | 1_2 | 0.2135 ± 0.0256 | 0.2396 ± 0.0285 | 2.12 × 10⁻⁸ | 5.72 × 10⁻⁸ | 0.516 |
| ≥160 | 1_3 | 0.1673 ± 0.0189 | 0.1868 ± 0.0198 | 3.69 × 10⁻⁹ | 1.42 × 10⁻⁸ | 0.543 |
| ≥160 | 1_5 | 0.1265 ± 0.0136 | 0.1409 ± 0.0143 | 2.70 × 10⁻⁹ | 1.42 × 10⁻⁸ | 0.548 |
| ≥160 | 1_6 | 0.1148 ± 0.0118 | 0.1280 ± 0.0130 | 1.75 × 10⁻⁹ | 1.42 × 10⁻⁸ | 0.555 |
| ≥160 | 1_8 | 0.0991 ± 0.0100 | 0.1102 ± 0.0110 | 2.22 × 10⁻⁹ | 1.42 × 10⁻⁸ | 0.551 |
| ≥160 | 1_10 | 0.0886 ± 0.0087 | 0.0984 ± 0.0097 | 1.58 × 10⁻⁹ | 1.42 × 10⁻⁸ | 0.556 |
| ≥160 | H2/H1 | 0.7107 ± 0.4003 | 0.4074 ± 0.2590 | 6.45 × 10⁻⁸ | 1.58 × 10⁻⁷ | −0.498 |
| ≥160 | LF/HF | 1.1227 ± 1.2891 | 0.4833 ± 0.5136 | 1.29 × 10⁻⁴ | 2.32 × 10⁻⁴ | −0.353 |
| ≥160 | Ts | 0.1889 ± 0.0305 | 0.2264 ± 0.0683 | 1.21 × 10⁻⁸ | 3.63 × 10⁻⁸ | 0.525 |
| ≥160 | Ts/Td | 0.6832 ± 0.1235 | 0.8532 ± 0.2744 | 3.35 × 10⁻⁹ | 1.42 × 10⁻⁸ | 0.545 |
| ≥160 | STD_AREA | 44.1992 ± 4.0982 | 47.8491 ± 4.8497 | 3.20 × 10⁻⁶ | 7.21 × 10⁻⁶ | 0.429 |

Values are presented as mean ± standard deviation. Between-group comparisons were performed using the Mann–Whitney U test. Rank-biserial correlation was used as the corresponding effect-size measure; positive values indicate higher feature values in participants aged ≥65 years, whereas negative values indicate lower values in the ≥65-year group. Benjamini–Hochberg false discovery rate correction was applied separately within each SBP stratum across all 27 PPG features included in the initial screening analysis. Physiologically implausible negative Ts and Ts/Td values were treated as invalid for analyses involving the corresponding timing features, resulting in n = 87 for the ≥65-year subgroup in the ≤120-mmHg stratum for these two variables. FDR, false discovery rate; LF, low frequency; HF, high frequency; Ts/Td, systolic-to-diastolic duration ratio; STD_AREA, standard deviation of beat-wise pulse area.
