# Supplementary material for: Age-associated photoplethysmographic features across systolic blood pressure strata: a cross-sectional study
Source: Front Physiol. 2026 Oct 29;17:1992759. doi: 10.3389/fphys.2026.1992759 (PMC13754651; doi:10.3389/fphys.2026.1992759)
Supplement: Supplementary file 4 [file Table4.docx]

**Supplementary Table S4. Complete Mann–Whitney U screening results for all 27 PPG-derived features across measured SBP strata**

| SBP stratum | Feature | <65, mean ± SD | ≥65, mean ± SD | P-value | FDR-adjusted q-value | Rank-biserial r |
| --- | --- | --- | --- | --- | --- | --- |
| ≤120 | 1_10 | 0.0829 ± 0.0090 | 0.0864 ± 0.0086 | 0.002 | 0.005 | 0.262 |
| ≤120 | 1_8 | 0.0927 ± 0.0102 | 0.0968 ± 0.0101 | 0.001 | 0.005 | 0.267 |
| ≤120 | 1_6 | 0.1073 ± 0.0118 | 0.1124 ± 0.0118 | 0.000791 | 0.004 | 0.277 |
| ≤120 | 1_5 | 0.1180 ± 0.0128 | 0.1237 ± 0.0132 | 0.000827 | 0.004 | 0.276 |
| ≤120 | 1_3 | 0.1568 ± 0.0177 | 0.1646 ± 0.0184 | 0.000777 | 0.004 | 0.277 |
| ≤120 | 1_2 | 0.2019 ± 0.0251 | 0.2119 ± 0.0262 | 0.003 | 0.007 | 0.247 |
| ≤120 | deltaT | 0.4384 ± 0.1120 | 0.4648 ± 0.1299 | 0.106 | 0.191 | 0.133 |
| ≤120 | P1 | 391.1689 ± 224.1598 | 411.8620 ± 248.2117 | 0.713 | 0.802 | 0.030 |
| ≤120 | P2 | 218.2113 ± 151.5799 | 222.6987 ± 160.0119 | 0.847 | 0.915 | -0.016 |
| ≤120 | Std_AREA | 41.1850 ± 5.1256 | 42.9635 ± 4.9725 | 0.002 | 0.005 | 0.257 |
| ≤120 | AC/DC | 0.3316 ± 0.2092 | 0.3310 ± 0.2004 | 0.921 | 0.966 | -0.008 |
| ≤120 | AMP | 207.0866 ± 114.1979 | 226.2909 ± 134.4381 | 0.498 | 0.654 | 0.056 |
| ≤120 | Ts | 0.1852 ± 0.0248 | 0.2078 ± 0.0758 | 0.000127 | 0.003 | 0.317 |
| ≤120 | Td | 0.2843 ± 0.0194 | 0.2834 ± 0.0192 | 0.543 | 0.654 | -0.050 |
| ≤120 | Ts/Td | 0.6550 ± 0.1018 | 0.7338 ± 0.2226 | 0.000515 | 0.004 | 0.287 |
| ≤120 | Ss | 2080.2980 ± 1157.8004 | 2017.4016 ± 1205.5201 | 0.557 | 0.654 | -0.048 |
| ≤120 | Ds | -382.4063 ± 328.6231 | -340.0180 ± 298.6586 | 0.155 | 0.262 | 0.117 |
| ≤120 | Area | 103.4998 ± 71.5030 | 114.5355 ± 82.9932 | 0.525 | 0.654 | 0.053 |
| ≤120 | H2/H1 | 0.7319 ± 0.4522 | 0.5917 ± 0.3466 | 0.040 | 0.077 | -0.169 |
| ≤120 | H3/H1 | 0.1536 ± 0.1323 | 0.1194 ± 0.1095 | 0.035 | 0.075 | -0.174 |
| ≤120 | H4/H1 | 0.0081 ± 0.0169 | 0.0070 ± 0.0147 | 0.966 | 0.966 | -0.004 |
| ≤120 | SDNN | 29.8402 ± 16.9689 | 27.4537 ± 15.1448 | 0.384 | 0.546 | -0.072 |
| ≤120 | RMSSD | 34.2473 ± 24.1989 | 36.6599 ± 22.1558 | 0.301 | 0.451 | 0.085 |
| ≤120 | PNN50 | 12.6116 ± 16.4241 | 16.0780 ± 17.6477 | 0.188 | 0.299 | 0.107 |
| ≤120 | LF | 0.0002 ± 0.0003 | 0.0001 ± 0.0004 | 0.007 | 0.016 | -0.224 |
| ≤120 | HF | 0.0003 ± 0.0005 | 0.0003 ± 0.0005 | 0.899 | 0.934 | 0.011 |
| ≤120 | LF/ HF | 0.8526 ± 0.8469 | 0.5919 ± 0.7204 | 0.000494 | 0.004 | -0.287 |
| 121–139 | 1_10 | 0.0847 ± 0.0105 | 0.0903 ± 0.0112 | 1.97e-07 | 5.90e-07 | 0.296 |
| 121–139 | 1_8 | 0.0948 ± 0.0118 | 0.1012 ± 0.0126 | 1.85e-07 | 5.90e-07 | 0.296 |
| 121–139 | 1_6 | 0.1098 ± 0.0140 | 0.1175 ± 0.0149 | 6.96e-08 | 2.69e-07 | 0.306 |
| 121–139 | 1_5 | 0.1209 ± 0.0156 | 0.1295 ± 0.0166 | 6.93e-08 | 2.69e-07 | 0.307 |
| 121–139 | 1_3 | 0.1603 ± 0.0214 | 0.1721 ± 0.0223 | 1.22e-08 | 8.22e-08 | 0.324 |
| 121–139 | 1_2 | 0.2056 ± 0.0289 | 0.2215 ± 0.0294 | 3.56e-09 | 3.21e-08 | 0.336 |
| 121–139 | deltaT | 0.4301 ± 0.1189 | 0.4629 ± 0.1171 | 0.003 | 0.005 | 0.169 |
| 121–139 | P1 | 423.3278 ± 277.6218 | 458.9304 ± 275.8163 | 0.201 | 0.236 | 0.073 |
| 121–139 | P2 | 231.9442 ± 169.6590 | 256.1116 ± 173.2168 | 0.166 | 0.204 | 0.079 |
| 121–139 | Std_AREA | 41.9495 ± 5.0815 | 44.1690 ± 5.1767 | 9.61e-05 | 0.000224 | 0.222 |
| 121–139 | AC/DC | 0.3335 ± 0.2095 | 0.3782 ± 0.2485 | 0.098 | 0.140 | 0.094 |
| 121–139 | AMP | 224.9371 ± 145.9522 | 246.5339 ± 146.1220 | 0.129 | 0.166 | 0.086 |
| 121–139 | Ts | 0.1923 ± 0.0865 | 0.2147 ± 0.0637 | 6.02e-14 | 1.62e-12 | 0.427 |
| 121–139 | Td | 0.2830 ± 0.0192 | 0.2786 ± 0.0197 | 0.034 | 0.052 | -0.120 |
| 121–139 | Ts/Td | 0.6880 ± 0.3637 | 0.7779 ± 0.2714 | 1.94e-12 | 2.62e-11 | 0.400 |
| 121–139 | Ss | 2239.3254 ± 1430.0797 | 2211.2728 ± 1320.6022 | 0.785 | 0.785 | -0.015 |
| 121–139 | Ds | -410.9276 ± 349.1104 | -415.1975 ± 381.1878 | 0.555 | 0.577 | 0.034 |
| 121–139 | Area | 110.7044 ± 81.6134 | 133.8298 ± 97.2097 | 0.010 | 0.016 | 0.146 |
| 121–139 | H2/H1 | 0.7391 ± 0.4907 | 0.4965 ± 0.2923 | 5.25e-08 | 2.69e-07 | -0.309 |
| 121–139 | H3/H1 | 0.1237 ± 0.1047 | 0.1063 ± 0.0968 | 0.069 | 0.098 | -0.103 |
| 121–139 | H4/H1 | 0.0094 ± 0.0264 | 0.0063 ± 0.0088 | 0.084 | 0.113 | 0.098 |
| 121–139 | SDNN | 29.8872 ± 20.8678 | 32.9724 ± 28.7991 | 0.316 | 0.341 | 0.057 |
| 121–139 | RMSSD | 36.4289 ± 31.6682 | 45.1829 ± 36.5959 | 0.000273 | 0.000566 | 0.207 |
| 121–139 | PNN50 | 14.1691 ± 18.6202 | 20.0685 ± 20.1491 | 0.00035 | 0.000675 | 0.201 |
| 121–139 | LF | 0.0002 ± 0.0003 | 0.0004 ± 0.0028 | 0.005 | 0.009 | -0.158 |
| 121–139 | HF | 0.0004 ± 0.0007 | 0.0008 ± 0.0032 | 0.267 | 0.300 | 0.063 |
| 121–139 | LF/ HF | 0.7872 ± 0.7382 | 0.5452 ± 0.6993 | 1.68e-06 | 4.54e-06 | -0.272 |
| 140–159 | 1_10 | 0.0869 ± 0.0098 | 0.0913 ± 0.0108 | 6.52e-05 | 0.000135 | 0.246 |
| 140–159 | 1_8 | 0.0973 ± 0.0111 | 0.1022 ± 0.0122 | 7.99e-05 | 0.000144 | 0.243 |
| 140–159 | 1_6 | 0.1129 ± 0.0132 | 0.1187 ± 0.0142 | 7.09e-05 | 0.000137 | 0.245 |
| 140–159 | 1_5 | 0.1243 ± 0.0147 | 0.1308 ± 0.0158 | 4.30e-05 | 0.000106 | 0.252 |
| 140–159 | 1_3 | 0.1651 ± 0.0203 | 0.1741 ± 0.0212 | 3.02e-05 | 8.16e-05 | 0.257 |
| 140–159 | 1_2 | 0.2118 ± 0.0273 | 0.2239 ± 0.0282 | 2.36e-05 | 7.07e-05 | 0.261 |
| 140–159 | deltaT | 0.4199 ± 0.0989 | 0.4726 ± 0.1112 | 1.19e-05 | 4.02e-05 | 0.270 |
| 140–159 | P1 | 423.9167 ± 285.6835 | 463.5725 ± 260.5462 | 0.031 | 0.042 | 0.133 |
| 140–159 | P2 | 245.9725 ± 193.7515 | 252.0476 ± 153.0984 | 0.146 | 0.164 | 0.090 |
| 140–159 | Std_AREA | 42.5847 ± 4.5096 | 44.8249 ± 5.1991 | 5.70e-05 | 0.000128 | 0.248 |
| 140–159 | AC/DC | 0.3448 ± 0.2435 | 0.3825 ± 0.2499 | 0.015 | 0.149 | 0.022 |
| 140–159 | AMP | 221.0248 ± 144.5243 | 254.3630 ± 145.6463 | 0.006 | 0.009 | 0.169 |
| 140–159 | Ts | 0.1910 ± 0.0383 | 0.2200 ± 0.0490 | 1.69e-14 | 4.57e-13 | 0.473 |
| 140–159 | Td | 0.2791 ± 0.0161 | 0.2809 ± 0.0209 | 0.759 | 0.759 | 0.019 |
| 140–159 | Ts/Td | 0.6879 ± 0.1494 | 0.7878 ± 0.1839 | 1.86e-10 | 2.51e-09 | 0.393 |
| 140–159 | Ss | 2199.8813 ± 1436.8744 | 2175.6323 ± 1191.0195 | 0.596 | 0.619 | 0.033 |
| 140–159 | Ds | -441.2934 ± 374.5212 | -376.1045 ± 307.6101 | 0.111 | 0.136 | 0.098 |
| 140–159 | Area | 115.7348 ± 94.2470 | 136.0621 ± 91.1134 | 0.001 | 0.002 | 0.197 |
| 140–159 | H2/H1 | 0.6657 ± 0.3701 | 0.4645 ± 0.2760 | 1.22e-08 | 1.10e-07 | -0.351 |
| 140–159 | H3/H1 | 0.1197 ± 0.1166 | 0.0960 ± 0.0736 | 0.166 | 0.180 | -0.085 |
| 140–159 | H4/H1 | 0.0051 ± 0.0096 | 0.0069 ± 0.0131 | 0.003 | 0.004 | 0.186 |
| 140–159 | SDNN | 27.1442 ± 13.8252 | 30.7093 ± 17.5721 | 0.040 | 0.051 | 0.127 |
| 140–159 | RMSSD | 32.3335 ± 19.9341 | 42.0991 ± 25.6421 | 3.56e-06 | 1.37e-05 | 0.286 |
| 140–159 | PNN50 | 11.3267 ± 12.7936 | 20.2994 ± 18.3299 | 1.79e-06 | 8.05e-06 | 0.293 |
| 140–159 | LF | 0.0002 ± 0.0004 | 0.0001 ± 0.0003 | 0.117 | 0.137 | -0.097 |
| 140–159 | HF | 0.0003 ± 0.0009 | 0.0004 ± 0.0008 | 0.002 | 0.002 | 0.195 |
| 140–159 | LF/ HF | 0.8510 ± 0.8085 | 0.5112 ± 0.5799 | 1.16e-07 | 6.26e-07 | -0.327 |
| ≥160 | 1_10 | 0.0886 ± 0.0087 | 0.0984 ± 0.0097 | 1.58e-09 | 1.42e-08 | 0.556 |
| ≥160 | 1_8 | 0.0991 ± 0.0100 | 0.1102 ± 0.0110 | 2.22e-09 | 1.42e-08 | 0.551 |
| ≥160 | 1_6 | 0.1148 ± 0.0118 | 0.1280 ± 0.0130 | 1.75e-09 | 1.42e-08 | 0.555 |
| ≥160 | 1_5 | 0.1265 ± 0.0136 | 0.1409 ± 0.0143 | 2.70e-09 | 1.42e-08 | 0.548 |
| ≥160 | 1_3 | 0.1673 ± 0.0189 | 0.1868 ± 0.0198 | 3.69e-09 | 1.42e-08 | 0.543 |
| ≥160 | 1_2 | 0.2135 ± 0.0256 | 0.2396 ± 0.0285 | 2.12e-08 | 5.72e-08 | 0.516 |
| ≥160 | deltaT | 0.3864 ± 0.1061 | 0.4173 ± 0.1153 | 0.083 | 0.112 | 0.160 |
| ≥160 | P1 | 503.8304 ± 304.1986 | 567.9929 ± 366.1953 | 0.328 | 0.385 | 0.090 |
| ≥160 | P2 | 278.2424 ± 193.0287 | 315.3535 ± 208.4707 | 0.193 | 0.248 | 0.120 |
| ≥160 | Std_AREA | 44.1992 ± 4.0982 | 47.8491 ± 4.8497 | 3.20e-06 | 7.21e-06 | 0.429 |
| ≥160 | AC/DC | 0.4004 ± 0.2430 | 0.4644 ± 0.2996 | 0.191 | 0.247 | 0.120 |
| ≥160 | AMP | 259.6266 ± 152.5306 | 300.5760 ± 203.6567 | 0.308 | 0.378 | 0.094 |
| ≥160 | Ts | 0.1889 ± 0.0305 | 0.2264 ± 0.0683 | 1.21e-08 | 3.63e-08 | 0.525 |
| ≥160 | Td | 0.2779 ± 0.0178 | 0.2675 ± 0.0180 | 0.000459 | 0.000774 | -0.323 |
| ≥160 | Ts/Td | 0.6832 ± 0.1235 | 0.8532 ± 0.2744 | 3.35e-09 | 1.42e-08 | 0.545 |
| ≥160 | Ss | 2660.7569 ± 1590.2104 | 2560.1063 ± 1656.9182 | 0.547 | 0.591 | -0.056 |
| ≥160 | Ds | -598.0203 ± 522.1208 | -566.5525 ± 436.6013 | 0.973 | 0.973 | -0.003 |
| ≥160 | Area | 126.6605 ± 89.8454 | 167.1768 ± 119.6027 | 0.028 | 0.040 | 0.202 |
| ≥160 | H2/H1 | 0.7107 ± 0.4003 | 0.4074 ± 0.2590 | 6.45e-08 | 1.58e-07 | -0.498 |
| ≥160 | H3H1 | 0.0864 ± 0.0712 | 0.0482 ± 0.0428 | 0.000536 | 0.000851 | -0.319 |
| ≥160 | H4/H1 | 0.0029 ± 0.0065 | 0.0031 ± 0.0070 | 0.361 | 0.406 | 0.084 |
| ≥160 | SDNN | 23.4641 ± 10.5697 | 33.8800 ± 23.4555 | 0.000109 | 0.00021 | 0.357 |
| ≥160 | RMSSD | 28.1126 ± 16.5423 | 48.6310 ± 35.5977 | 5.87e-09 | 1.98e-08 | 0.536 |
| ≥160 | PNN50 | 8.6681 ± 13.4161 | 23.3187 ± 17.3740 | 2.64e-09 | 1.42e-08 | 0.546 |
| ≥160 | LF | 0.0001 ± 0.0001 | 0.0002 ± 0.0006 | 0.917 | 0.952 | -0.010 |
| ≥160 | HF | 0.0002 ± 0.0002 | 0.0007 ± 0.0025 | 6.73e-05 | 0.00014 | 0.367 |
| ≥160 | LF/ HF | 1.1227 ± 1.2891 | 0.4833 ± 0.5136 | 0.000129 | 0.000232 | -0.353 |
